# Heparin Immobilization Enhances Hemocompatibility, Re-Endothelization, and Angiogenesis of Decellularized Liver Scaffolds

**DOI:** 10.3390/ijms252212132

**Published:** 2024-11-12

**Authors:** Chandra Jit Yadav, Usha Yadav, Sadia Afrin, Jun-Yeong Lee, Jihad Kamel, Kyung-Mee Park

**Affiliations:** College of Veterinary Medicine, Chungbuk National University, Cheongju 28644, Republic of Korea; chandrajityadav84@gmail.com (C.J.Y.); ushacj.23@gmail.com (U.Y.); sadiaafrin1406@gmail.com (S.A.); dkujunyeong@naver.com (J.-Y.L.); jihadshabaan@gmail.com (J.K.)

**Keywords:** liver, heparin, hemocompatibility, re-endothelialization, angiogenesis

## Abstract

Bioengineered livers are currently an acceptable alternative to orthotopic liver transplants to overcome the scarcity of donors. However, the challenge of using a bioengineered liver is the lack of an intact endothelial layer in the vascular network leading to thrombosis. Heparin-modified surfaces have been demonstrated to decrease thrombogenicity in earlier research. However, in our study, we aimed to apply heparin immobilization to enhance the hemocompatibility, endothelial cell (EC) adhesion, and angiogenesis of rat decellularized liver scaffolds (DLS). Heparin was immobilized on the DLS by the end-point attachment technique. The scaffold’s hemocompatibility was assessed using ex vivo blood perfusion and platelet adhesion studies. The heparinized scaffold (HEP-DLS) showed a significantly reduced thrombogenicity and platelet aggregation. HEP-DLS was recellularized with EA.hy926 cells via the portal vein and maintained in the bioreactor for 7 days, showing increased EC adhesion and coverage within the blood vessels. The Resazurin reduction assay confirmed the presence of actively proliferating cells in the HEP-DLS. The scaffolds were implanted subcutaneously into the dorsum of mice for 21 days to evaluate cell migration and angiogenesis. The results showed significant increases in the number of blood vessels in the HEP-DLS group. Our results demonstrated that heparin immobilization reduces thrombosis, promotes re-endothelialization, and enhances angiogenesis in DLS. The research provides insight into the potential use of heparin in the formation of a functioning vasculature.

## 1. Introduction

Liver transplantation is the only curative treatment available for end-stage liver disease (ESLD) [1]. The shortage of healthy donor livers creates a substantial gap between patients on the waiting list and available organs, leading to a high mortality rate [2]. In this scenario, a bioengineered liver is a potential alternative for solving the shortage of liver transplant donors. Liver tissue engineering is a recently emerged approach for the development of decellularized liver scaffolds (DLS) in generating a functional liver.

The process of perfusion decellularization is an effective method for producing whole-organ scaffolds [3]. Liver scaffolds that have been decellularized retain their microstructure, biomechanical characteristics, and bioactive signals, making them suitable for recellularization [4,5]. However, the recellularization process remains challenging, and many hurdles must be addressed prior to the practical application of recellularized liver transplantation. During the overall decellularization process, the key extracellular matrix (ECM) components and ultrastructure were susceptible to loss and degradation, which will impair the adherence and proliferation of re-seeded cells. Vascular patency is one of the most major obstacles to successful organ bioengineering [6,7]. The endothelial cell (EC) lining of blood vessels is required to provide a non-thrombotic barrier within decellularized organs [8].

Recent research has focused on the surface modification of decellularized scaffolds to enhance the attachment of ECs to the inner lining of blood vessels for the development of bioengineered livers. Various strategies have been implemented and optimized to re-establish functional vasculature in decellularized scaffolds, such as the application of anti-endothelial cell antibodies [9], heparin–gelatin [10], anti-CD31 aptamers [11], and REDV peptides [12].

EC seeding has the potential to reduce coagulation. However, it frequently results in an incomplete coverage of the vasculature, leaving thrombogenic defects in the vascular lining. In tissue engineering, one strategy to improve transplant hemocompatibility is surface modification using bioactive chemicals, such as heparin [13,14]. Heparin is a widely used anticoagulant. Heparin-modified scaffold collagen has been shown to prevent blood clot formation [15,16]. Heparin, covalently immobilized on the surface of biomaterials via an end point, has been demonstrated to retain its anti-thrombogenic activity [17]. The scaffold heparinized via the end-point attachment (EPA) technique exhibited increased heparin immobilization efficiency, resulting in enhanced hemocompatibility and biocompatibility [18]. Heparin can bind to ECs and exhibits non-thrombogenic characteristics [19,20]. Heparin’s strong affinity for ECs is due to its high negative charge density from sulfate and carboxyl groups on its polysaccharide chains [21], facilitating electrostatic interactions with positively charged domains on EC surfaces [20]. Additionally, heparin interacts with growth factors to promote EC proliferation [22], which is essential for angiogenesis. Previous studies have demonstrated that heparin and heparin-like polymers can significantly promote angiogenesis and reduce the need for exogenous growth factors [23]. Both in vitro and in vivo studies have shown that heparinized scaffolds effectively recruit and localize endogenous factors to locally increase vessel density, showing the angiogenic potential [24]. However, the efficacy of heparin modification in ensuring the functional impact within entire liver ECM scaffolds remains still uncertain.

Heparin is a multifunctional bioactive molecule that not only serves as an anticoagulant but also possesses the ability to bind with EC, as well as promote angiogenesis. Therefore, in this study, we modify the decellularized whole rat liver scaffold through heparin immobilization using EPA techniques. This study aims to develop and evaluate a heparinized decellularized liver scaffold (HEP-DLS) as a viable strategy for overcoming the challenge of establishing functional vasculature in liver tissue engineering. We hypothesize that the immobilization of heparin to DLS will improve hemocompatibility, enhance EC attachment, promote re-endothelialization, and stimulate angiogenesis, ultimately leading to the better integration and functionality of engineered liver tissues.

## 2. Results

### 2.1. Characterization of the Decellularized Rat Liver Scaffold

**Figure 1:**
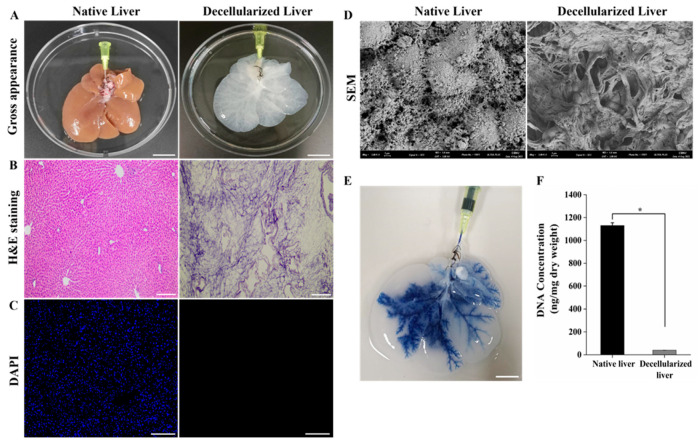
Decellularization of rat liver. (**A**) Gross appearance of decellularized liver appears as a translucent white color and retains its gross anatomical features (Scale bar = 2 cm). (**B**,**C**) H&E and DAPI staining of decellularized liver showing complete removal of cellular matrix compared to native liver and maintenance of 3D architecture (Scale bar = 200 μm). (**D**) SEM image of decellularized liver exhibits no residual cells and well-preserved extracellular matrix (Scale bar = 2 μm). (**E**) Trypan blue dye perfusion through PV of decellularized scaffold showing the intact vasculature tree (Scale bar = 2 cm). (**F**) DNA quantification shows that the DNA content of DLS was significantly lower < 50 ng/mg (*n* = 5, * *p* < 0.05), confirming the efficiency of the decellularization.

A completely decellularized rat liver was obtained by perfusing 1% Triton X-100/0.1% ammonium hydroxide solution through the portal vein (PV). Macroscopically, the liver appeared with a translucent whitish color after the decellularization process, retaining the volume and shape of the native liver (Figure 1A). H&E and DAPI staining confirmed the complete removal of cells, while the scaffolds preserved the ECM structure and vascular structures (Figure 1B,C). Moreover, scanning electron microscopy (SEM) analysis provided visual confirmation of the successful removal of cells and illustrated the preservation of ultrastructural components in the decellularized liver (Figure 1D). The perfusion of trypan blue dye through the PV revealed the morphological vascular integrity, suggesting the preservation vasculature of DLS (Figure 1E). The DNA content of the DLS (40.09 ± 0.78) is significantly lower than that of the normal liver (1130.07 ± 24.74 ng/mg), indicating the removal of cellular components in the DLS (* *p* < 0.05) (Figure 1F). Thus, our study collectively validates the efficacy of the decellularization procedure, ensuring the elimination of cellular components while preserving the essential structural integrity of the liver scaffold. This framework provides an appropriate microenvironment for reseeding scaffolds.

### 2.2. Characterization of Heparinized DLS

**Figure 2:**
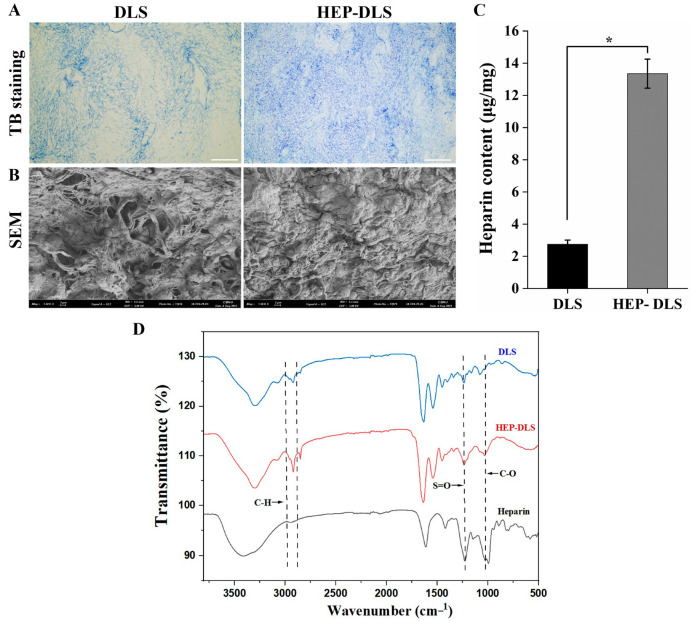
Heparin immobilization of rat decellularized liver through EPA techniques. (**A**) Histological toluidine blue staining of the DLS and HEP-DLS. Blue staining of HEP-DLS confirms the heparin immobilization (scale bar = 200 μm). (**B**) SEM of HEP-DLS displays a distinct uniform microtopography (Scale bar = 2 μm). (**C**) Quantitative analysis of heparin content of DLS and HEP-DLS by toluidine blue O assay *(n* = 4, * *p* < 0.05). (**D**) FTIR spectra of DLS, HEP-DLS, and heparin.

We confirmed the heparin immobilization through toluidine blue (TBO) staining, revealing pervasive blue staining across the ECM of the HEP-DLS, while the non-heparinized DLS remained unstained (Figure 2A). A SEM image of the HEP-DLS displayed a distinctive uniform microtopography throughout the entire matrix, compared to the DLS (Figure 2B). Furthermore, a quantitative analysis employing a TBO colorimetric assay indicated a heparin content of 13.53 ± 0.90 µg/mg in the HEP-DLS, while the DLS contained significantly lower heparin of 2.75 ± 0.25 µg/mg, as shown in Figure 2C. Further elucidation of heparinization was obtained through Fourier-transform infrared spectroscopy (FTIR). The FTIR spectra of DLS, HEP-DLS, and pure heparin are presented in the accompanying Figure 2D. The signals at 1043 cm^−1^ (C-O) and 1220 cm^−1^ (S=O) in the spectra of HEP-DLS corresponded with the heparin signal, which confirmed that heparin was covalently immobilized to the scaffolds.

### 2.3. Anticoagulant Efficacy of Heparin Modified Scaffolds

**Figure 3:**
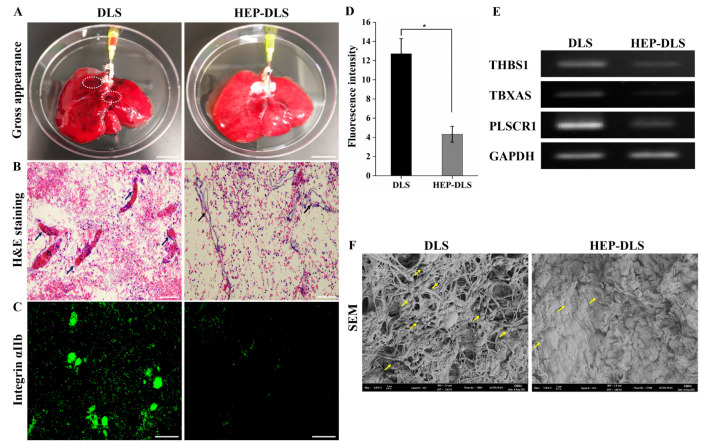
Ex vivo blood perfusion and platelets adhesion test. (**A**) The gross morphology of DLS and HEP-DLS after 40 min of blood perfusion. DLS shows multiple dark blood clots, as indicated in circle (Scale bar = 2 cm). (**B**) H&E showed thrombosis in vasculature of blood-perfused DLS (arrows), whereas no clots in HEP-DLS (scale bar = 100 μm). (**C**) Immunofluorescence staining with anti-integrin αIIb indicates platelet adhesion on scaffolds (scale bar = 100 μm). (**D**) Quantification of fluorescence intensity of integrin αIIb expression. Each group (*n* = 4), mean ± SD, * *p* < 0.05. (**E**) PCR analysis showing low expressions of thrombogenicity-related genes, THBS1; thrombospondin, TBXAS; thromboxane A synthase, PLSCR1; phospholipid scramblase in blood perfused HEP-DLS compared to DLS. (**F**) SEM image reveals substantial platelet aggregation and adhesion across the surface of non-heparinized DLS compared to HEP-DLS. Yellow arrows indicate adherent platelets (scale bar = 2 μm).

To evaluate the potential of HEP-DLS for enhancing hemocompatibility, both DLS and HEP-DLS scaffolds were perfused with diluted porcine whole blood for 40 min. A gross observation showed extensive dark blood clots in the non-heparinized DLS, while the HEP-DLS exhibited no clot formation (Figure 3A). Additionally, H&E staining indicated that the vascular structures in the DLS were occluded by thrombus, whereas the vasculature in the HEP-DLS remained clear (Figure 3B). Immunostaining with anti-integrin αIIb revealed significant platelet adhesion and aggregation within the vascular structure and matrix of the DLS. In contrast, the heparinized scaffolds showed markedly fewer platelets (Figure 3C). These findings suggest that HEP-DLS improves the hemocompatibility of scaffolds. A polymerase chain reaction (PCR) analysis revealed a high expression of thrombogenicity-related genes (THBS1, TBXAS1, and PLSCR1) in the blood-perfused DLS compared to the HEP-DLS (Figure 3E). Furthermore, platelet adhesion studies were conducted to determine the thrombogenicity of heparinized scaffolds. SEM images revealed a minimal platelet presence on the surface of the heparinized scaffold, while the non-heparinized group showed extensive platelet aggregation and adhesion (Figure 3F). Overall, these results demonstrate that the heparin-modified scaffolds function as non-thrombogenic constructs, effectively inhibiting platelet attachment.

### 2.4. Structural Characterization of Re-Endothelialized Scaffold

**Figure 4:**
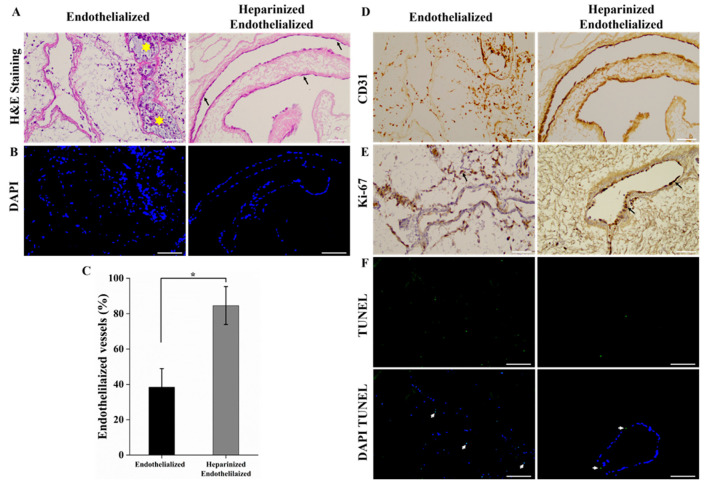
Re-endothelialization of decellularized and heparin-modified decellularized liver using EA.hy926 cells for 7 days. (**A**,**B**) H&E and DAPI staining of the heparinized re-endothelialized scaffold shows more ECs adhered to the vessels (black arrows) while ECs escaped to the parenchyma and obstructed vessel lumen (yellow stars) in case of endothelialized scaffold (control) (scale bar = 100 μm). (**C**) Significantly higher percentage of endothelialized vessels per field was observed in heparinized re-endothelialized scaffold from different five lobes, *n* = 3 field/slide, * *p* < 0.05. (**D**) IHC staining of ECs with CD31 antibody. (**E**) Ki-67 staining confirms EC proliferation after cell seeding into the scaffolds. (scale bar = 100 μm) (**F**) TUNEL assay shows fewer numbers of apoptotic cells in heparinized group than control (white arrow indicates the apoptotic cells) (scale bar = 100 μm).

In this study, we modified DLS with heparin to enhance EC attachment to the vasculature. Following heparin modification, ECs were seeded in the scaffolds, cultured for seven days, and subsequently harvested for histological analysis. H&E and DAPI staining revealed that, in HEP-DLS, ECs showed robust attachment to the vasculature, forming a well-defined EC lining within the blood vessels (Figure 4A,B). In contrast, in the re-endothelialized scaffold without heparin immobilization (control), no vessel lining was observed, and the ECs were dispersed in the parenchymal areas. The control group exhibited vessel occlusion with ECs obstructing the vessel lumen (indicated by a yellow star). Quantitative analysis demonstrated that approximately 84.59 ± 10.71% of the scaffold vasculature in the heparinized group was covered by ECs, compared to only 38.43 ± 10.51% in the uncoated scaffolds (Figure 4C). For an overview of the endothelialized vessels, H&E staining images of different lobes of the re-endothelialized scaffolds are provided in Supplementary Figure S1. An immunohistochemical (IHC) analysis detected CD31 expression on the inner surfaces of vessels throughout the heparinized re-endothelialized liver scaffolds (Figure 4D). To evaluate the proliferation ability of reseeded ECs in the re-endothelialized liver scaffolds, Ki67 staining was performed. The results indicated that the majority of cells were proliferative in the heparinized group (Figure 4E). Additionally, TUNEL staining exhibited a lower level of apoptotic cells in the heparinized group relative to the non-coated group (Figure 4F). These findings underscore the pivotal role of heparin immobilization in facilitating the attachment of EC to the vasculature structure and enhancing the re-endothelialization process of the scaffolds.

### 2.5. Evaluation of Functionality of Re-Endothelialized Liver Scaffolds

**Figure 5:**
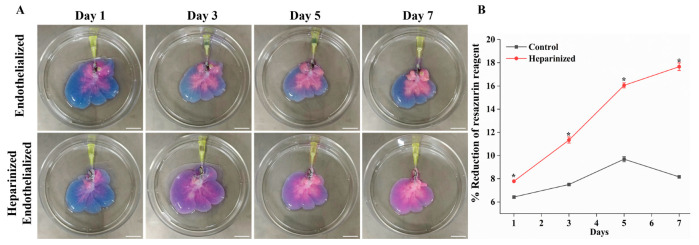

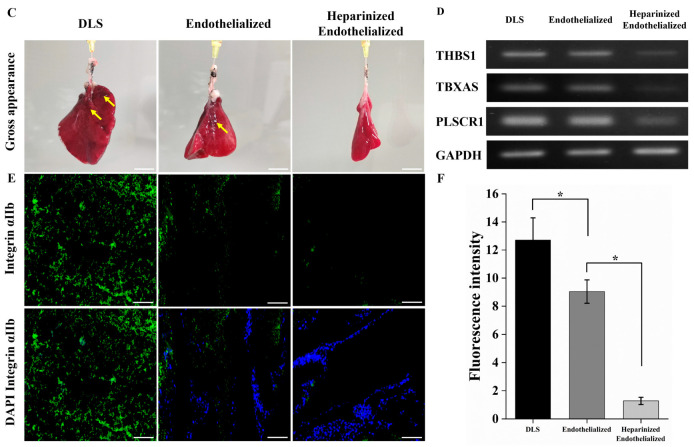
Resazurin reduction assay and ex vivo blood perfusion of re-endothelialized scaffold. (**A**) Resazurin reduction assay: visual photograph showing reduction of resazurin reagent from blue to pink over time, indicating cell proliferation in control and heparin-modified re-endothelialized liver scaffolds (Scale bar = 2 cm). (**B**) The curve shows significant proliferation of cells in heparinized re-endothelialized scaffolds compared to control (*n* = 3, * *p* < 0.05). (**C**) Gross appearance of heparinized re-endothelialized scaffold was free of clots compared to the non-coated re-endothelialized and DLS (yellow arrows indicate clot) (scale bar = 2 cm). (**D**) PCR analysis showed low expressions of thrombogenicity-related genes (THBS1; thrombospondin 1, TBXAS; thromboxane A synthase, PLSCR1; phospholipid scramblase 1) in blood-perfused heparinized re-endothelialized scaffolds compared to re-endothelialized and decellularized scaffolds. (**E**) Immunofluorescence staining with anti-integrin αIIb (green) and DAPI (blue) showing the platelets adherence and EC attachment in the scaffolds (scale bar = 100 μm). (**F**) Quantification of fluorescence intensity of integrin αIIb (green) demonstrates a significant reduction in intensity of heparin-treated re-endothelialized scaffolds, *n* = 4 fields, * *p* < 0.05.

In our study, we utilized the Resazurin reduction perfusion assay to evaluate the viability and proliferation of reseeded ECs on liver scaffolds. A resazurin–medium working solution (1:10 dilution) was perfused through the scaffolds at a flow rate of 2 mL/min for one hour on days 1, 3, 5, and 7 of recellularization. The photograph of the re-endothelialized liver scaffolds showed a gradual color change from blue to pink, indicating the metabolism of resazurin by viable cells within the constructs. Importantly, we observed a more intense color change in the heparinized re-endothelialized scaffolds each day of perfusion, suggesting increased cell attachment and proliferation (Figure 5A). The results shown in Figure 5B indicate that the percentage reduction of resazurin in heparinized re-endothelialized scaffolds (*n* = 3) increased to 7.79 ± 0.05% compared to 6.43 ± 0.09% in untreated scaffolds after one day of culture, indicating a higher number of cells attached to the heparinized scaffold. Over the course of 7 days of re-endothelialization, a significant increase in the percentage reduction of the resazurin reagent was observed in the re-endothelialized liver scaffolds immobilized with heparin, demonstrating rapid cell proliferation. In contrast, the control scaffolds did not show significant growth in the cell population and exhibited slight decreases in the resazurin reduction percentage by day 7. Overall, HEP-DLS facilitated greater EC attachment, suggesting efficient re-endothelialization.

In order to investigate the thrombogenicity of re-endothelialized constructs, porcine whole blood was perfused to the scaffold. Gross images of the re-endothelialized constructs after blood perfusion revealed that heparin-immobilized constructs showed no thrombosis, whereas endothelialized scaffolds and DLS (non-endothelialized) exhibited distinct thrombus formation, as indicated by yellow arrows (Figure 5C). A PCR analysis of the blood perfusion demonstrated elevated expression levels of thrombogenicity-related genes (THBS1, TBXAS, and PLSCR1) in blood-perfused decellularized scaffolds compared to re-endothelialized scaffolds. Heparin-modified endothelialized scaffolds exhibited a low expression of thrombogenic-related genes (Figure 5D). Immunostaining for integrin α IIb, expressed by activated platelets, showed substantial platelet aggregation in DLS without re-endothelialization and endothelialized scaffolds (Figure 5E,F). In contrast, heparin-modified re-endothelialized scaffolds showed fewer aggregated platelets. Overall, these findings suggest that heparin immobilization facilitates EC attachment, promoting the formation of a uniform and functional EC lining that inhibits platelet activation and adhesion to vessel walls.

### 2.6. In Vivo Angiogenesis Assessment of Modified Scaffold

**Figure 6:**
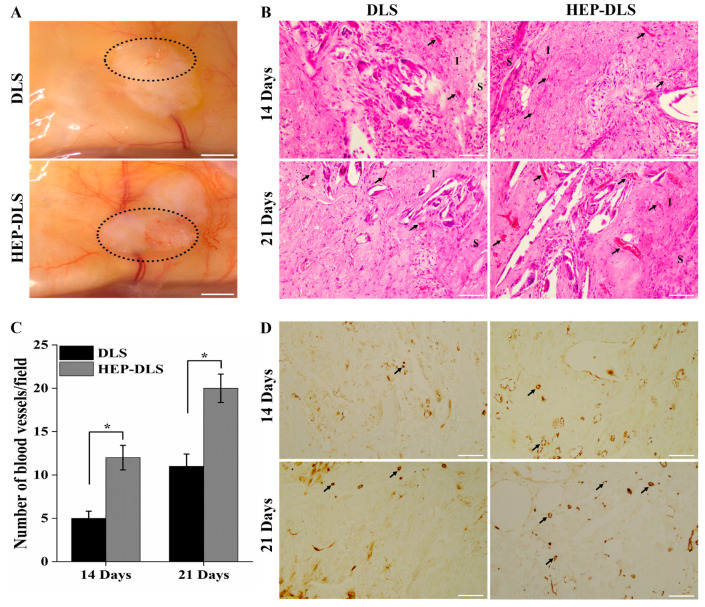
Angiogenic capability of DLS and HEP-DLS in vivo. (**A**) Gross appearances of scaffolds after 21 days of subcutaneous implantation in mice. Black circles indicate the neo-vessel formation within the scaffold (scale bar = 2 cm). (**B**) H&E staining showed more blood vessel formation (black arrows) in the HEP-DLS on 14 and 21 days post-implantation (scale bar = 100 μm); I, implanted areas; S, surrounding areas. (**C**) The number of blood vessels in each group of implants was expressed as the average per field on 14 and 21 days post-implantation (*n* = 4), * *p* < 0.05. (**D**) IHC staining of CD31 for the scaffolds on days 14 and 21 post-implantation, new blood vessels formed within the scaffolds (black arrows), (scale bar = 100 μm).

To evaluate the impact of HEP-DLS on the angiogenic potential, both DLS and HEP-DLS scaffolds without endothelialization were implanted on the dorsum of mice for a duration of 3 weeks. The scaffolds were harvested at the 2-week and 3-week points of post-implantation. No signs of inflammation or infection were observed in the mice, indicating a good biocompatibility of the implanted scaffold. The gross examination of the HEP-DLS scaffolds revealed a pronounced emergence of new blood vessels compared to the fewer blood vessels observed in the DLS scaffolds (Figure 6A). This observation was supported by H&E staining, which showed a significant increase in the number of blood vessels within the HEP-DLS group at both 14 and 21 days. In contrast, only a small number of blood vessels were observed in the DLS group (Figure 6B). A quantitative analysis of the blood vessel density demonstrated that the neovascularization in the HEP-DLS group was significantly higher than in the DLS group at both time points (* *p* < 0.05) (Figure 6C). CD31-positive cells indicate the recruitment and integration of host endothelial cells into the implanted scaffold. Heparinized scaffolds effectively recruit endogenous angiogenic factors from the host tissue, enhancing new blood vessel formation and demonstrating their angiogenic potential. These results highlight the potent angiogenic effects of HEP-DLS, indicating their potential as a favorable substrate for promoting vascularization in vivo.

## 3. Discussion

The development of transplantable bioengineered livers is being considered as a solution to the organ transplant shortage. DLS, primarily composed of ECM, provides an optimal microenvironment for cell adhesion, migration, differentiation, and metabolism through cell–matrix interactions [25]. Previous research demonstrated that using a solution of 1% Triton X-100/0.1% ammonium hydroxide for liver decellularization is advantageous. It effectively preserves ECM integrity, maintains the tissue’s ultrastructure, and minimizes ECM protein disruption [26,27]. Following this established procedure, we applied the same 1% Triton X-100/0.1% ammonium hydroxide solution to decellularize the liver, resulting in a translucent liver matrix with maintained vascular structures and a three-dimensional ECM architecture, confirming efficient decellularization. Bioengineered grafts offer a novel solution for constructing substitutes. However, their application is limited by thrombogenicity. The exposure of collagen in these grafts triggers coagulation upon blood reperfusion, posing a significant challenge [28]. Additionally, the vascularization of engineered tissues is also a critical step to prevent thrombosis and platelet adherence, ensuring the functionality of bioengineered livers [29]. Therefore, there is a demand to improve the hemocompatibility and re-endothelialization of scaffolds to prevent early thrombosis after implantation. The immobilization of heparin offers several advantages, such as being an anticoagulant, strong affinity to EC due to its negative charges, and promotion of angiogenesis which reduces the need for exogenous growth factors [17,18,19,21,23].

This study aimed to enhance the hemocompatibility of DLSs by modifying their surface with heparin. Furthermore, this modification was intended to improve EC binding to the vascular structures, enabling improved endothelialization and increasing the scaffold’s angiogenic potential. Heparin can be immobilized in various ways, including ionic binding and covalent binding through multipoint or EPA. Previous studies have highlighted the benefits of using the EPA method to enhance the blood and cell compatibility of DLS [18,30]. In this study, we applied the EPA method to immobilize heparin on DLSs.

In our study, we utilized whole blood perfusion in vitro as a model for reperfusion to investigate the thrombogenicity of HEP-DLS under controlled conditions. The findings revealed that heparin-immobilized liver scaffolds effectively prevent clot formation during ex vivo blood perfusion, thereby enhancing the scaffold’s hemocompatibility. These results align with previous reports indicating that heparin-immobilized scaffolds significantly reduce thrombogenicity [31,32]. Specifically, in our experiments, HEP-DLS maintained blood flow throughout the 40-min observation period, while the untreated scaffolds became completely obstructed with blood clots within 10 min. However, it was noted that the blood flow rate in the HEP-DLS decreased from inflow to outflow after 30 min, due to the absence of an endothelial lining in the vasculature. Based on this observation, we decided to perfuse blood for 40 min only. The scaffolds immobilized with heparin significantly inhibited platelet adhesion, as verified by SEM. These findings indicate that heparin pre-treatment of a DLS can effectively minimize thrombus formation and improve hemocompatibility.

However, for practical applications, endothelial coverage of the vascular lumen is crucial to prevent thrombosis and enhance vascular function [10,11,33]. Re-endothelialization enhances graft hemocompatibility by concealing the thrombogenic basement membrane for sustained anti-thrombotic capability. Heparin not only promotes an antithrombotic potential but also supports the adhesion and growth of reseeded cells [34]. Additionally, heparin’s negative charge density facilitates binding with ECs.

In this study, we successfully improved re-endothelialization efficiency by modifying the DLS with immobilized heparin. While various surface modifications, such as anti-endothelial cell antibodies [9], heparin–gelatin mixtures [10], gelatin [33], and REDV peptides [12], have been explored to enhance EC adhesion and promote the formation of a confluent endothelial layer, these methods mainly focus on cell attachment without directly improving scaffold hemocompatibility. Our results show that immobilizing heparin not only enhances hemocompatibility but also significantly accelerates EC recruitment and boosts the scaffold’s angiogenic potential. This suggests that heparin modification provides a more supportive environment for EC, facilitating both initial adhesion and the subsequent processes needed for the complete and functional re-endothelialization of the scaffolds. Our results demonstrated significant EC attachment and proliferation in the heparin-modified scaffold, resulting in a uniform cell lining within the vascular lumen. These findings are consistent with those of Hussein et al., who used a heparin–gelatin mixture for the endothelialization of porcine liver scaffolds [10]. Notably, we also observed that EC in the heparin-treated, re-endothelialized liver exhibited an elongated morphology, a characteristic of EC under physiological conditions, indicating effective EC attachment, similar to the results reported by Kim et al. [11].

The Resazurin reduction perfusion assay is an effective method for monitoring cell viability during the optimization of in vitro culture conditions [35,36]. In our study, we employed this assay to assess the viability and proliferation of reseeded cells within the scaffold during perfusion culture. We observed a significant increase in cell proliferation within the heparinized re-endothelialized scaffold over the first five days, followed by a subsequent slowdown in the proliferation rate. Unfortunately, a reduction in the size of the re-endothelialized scaffold was also noted after five days. We hypothesize that this decrease in cell proliferation could be attributed to the degeneration of the scaffold during prolonged culture. There is a possibility that the scaffold’s mechanical properties weakened, leading to the loss of some cells into the media. Another possible explanation might be a decline in the cell proliferative capacity, which could potentially be mitigated by the application of growth factors. Future studies will focus on applying the scaffold with reinforced mechanical properties. Moving forward, our research will aim to enhance the physical strength of the scaffold through cross-linking prior to recellularization. This approach is expected to improve the scaffold’s stability and support sustained cell proliferation.

Our study demonstrated the potential of improving functional endothelialization in the decellularized rat liver, as shown through ex vivo blood perfusion experiments. Endothelializing the tubular structures effectively prevents blood cells from leaking into the surrounding tissue [33,37]. We also observed that perfused blood predominantly followed the vasculature of the heparinized re-endothelialized liver scaffolds, with very few leakage points. This indicates that the ECs successfully concealed the thrombogenic basement membrane, leading to a significant reduction in platelet activation and aggregation within the scaffold. These results are similar to those reported by Kim et al. and Devalliere et al. who found that the efficient re-endothelialization of liver scaffolds reduced platelet adhesion and activation [11,12]. Thus, our findings confirm that re-endothelialization is a promising approach to enhancing the hemocompatibility of liver constructs, making them more viable for transplantation.

The covalent incorporation of heparin into collagen matrices has been explored to enhance their angiogenic potential, as documented in prior research [38,39]. Wu et al. have demonstrated that heparinized scaffolds exhibit angiogenic properties both in vitro and in vivo [24]. In our study, we employed covalent cross-linking of heparin onto DLS to promote vascularization. After harvesting the implanted scaffolds at 14 and 21 days post-implantation, we observed an increase in the formation of new blood vessels. These findings suggest that HEP-DLS effectively recruited and localized endogenous factors, thereby augmenting vessel density in the scaffold.

Heparin-modified surfaces have been proven to possess anticoagulant properties that exhibit strong hemocompatibility and biocompatibility profiles and facilitate angiogenesis in in vitro and in vivo studies [40]. Overall, our study produced functional constructs with improved hemocompatibility, which is attributed to heparin immobilization. However, there were some limitations. The heparin-modified, re-endothelialized constructs were not subjected to heterotopic transplantation, preventing us from assessing the graft’s patency. The engineering of whole-organ liver tissue is inherently complex, requiring numerous technically demanding steps to be executed sequentially. To move closer to creating a fully functional whole liver, our future research will incorporate both parenchymal and ECs into HEP-DLS. We will explore various seeding strategies to evaluate the impact of endothelialization on hepatocyte function in vivo. This approach aims to address the current limitations and advance the development of transplantable liver constructs.

## 4. Materials and Methods

### 4.1. Liver Harvest and Decellularization

Rat livers were decellularized as by the previously described method [26] with some modifications. Livers were harvested from 8-week-old female SD rats (Nara-Biotec, Seoul, Republic of Korea) weighing 250–300 g. Briefly, rats were anesthetized using Alfaxan (Zoetis, NJ, USA) 30 mg/kg and Domitor (Orion Pharma, Espoo, Finland) 0.5 mg/kg, and a U-shaped incision was made to expose the liver. The PV was cannulated with a 24 G catheter and perfused with 30 mL of heparinized phosphate-buffered saline (PBS) at a concentration of 10 IU/mL. The liver was then dissected and perfused with PBS for 60 min at 2 mL/min using a peristaltic pump (Jenie Well, Seoul, Republic of Korea). This was followed by an 8 h perfusion with 1% Triton X-100/0.1% ammonium hydroxide (SamChun, Seoul, Republic of Korea) at 3 mL/min. Finally, the liver was perfused with PBS for 16 h, sterilized with 0.1% peracetic acid, and stored at 4 °C in PBS containing antibiotics. The schematic diagram of the whole experiment is shown in Supplementary Figure S2. The experiments were approved by the Chungbuk National University Institutional Animal Center and Use Committee (CBNUA-2041-22-02).

### 4.2. Heparin Immobilization of DLS

Heparin was immobilized on the scaffold via EPA using a method modified from Bao et al. [18]. First, sodium heparin (H3149; Sigma-Aldrich, St. Louis, MO, USA) was solubilized in distilled water (DW) (1 g/300 mL) at 0 °C, then partially depolymerized by adding sodium nitrite (10 mg) (pH 2.7, adjusted with 1 N HCl) and stirring at 0 °C for 2 h. The pH of the solution was adjusted to 7.0 with 1 N NaOH. The solution containing partially depolymerized heparin with sodium cyanoborohydride (NaBH3CN, 0.01 mg/mL) and NaCl (0.15 M) at pH 3.5 was perfused into DLS for 4 h. Subsequently, DW was perfused at the rate of 2 mL/min for 4 hr. with hourly changes. Finally, the DLS was stored in PBS containing antibiotics at 4 °C until use.

### 4.3. Characterization of Heparin-Immobilized DLS

Formalin-fixed HEP-DLS and non-heparinized DLS tissue sections were stained with 1% toluidine blue (TBO) to determine the immobilization of heparin to scaffolds. The heparin content of the modified DLSs was determined using a TBO colorimetric method partially modified from Smith et al. [41]. In brief, the heparin-immobilized scaffold was incubated in 5 mL of a freshly prepared solution of 0.04 wt.% TBO in aqueous 0.01M HCl/0.2 wt.% NaCl. The samples were gently shaken at 37 °C for 4 h, then rinsed twice with DW, forming the Hep/TBO complex on the scaffold surface. Following this, 5 mL of a 4:1 (*v*/*v*) mixture of ethanol and 0.1 M NaOH were added to dissolve the Hep/TBO complex. After complete dissolution, 200 μL of the supernatant were added to a 96-well plate, and the OD value was measured at 530 nm using a microplate reader. The OD value was then used to calculate the amount of immobilized heparin from a standard heparin calibration curve. Furthermore, FTIR was used to detect immobilized heparin on the surface of the DLS.

### 4.4. Anticoagulation Assay and Platelets Adhesion Test

To assess the scaffold thrombogenicity ex vivo, whole blood perfusion was performed. The HEP-DLS and DLS were perfused with porcine blood obtained from a local slaughterhouse and treated with sodium citrate anticoagulant diluted with PBS (1:1) for 40 min. After perfusion with blood, scaffolds were flushed with PBS and observed macroscopically to determine the presence of blood clots. The scaffolds were fixed and processed for immunofluorescence analysis. In addition, perfused blood samples were collected for RNA isolation using the Hybrid-RTM Blood RNA kit (GeneAll Bio, Seoul, Republic of Korea) to determine thrombogenicity. cDNA was synthesized from the extracted RNA and subsequently amplified via PCR using primers targeting thrombogenic genes. The sequences of the primers used are listed in Table 1.

Platelet-rich plasma (PRP) was obtained by centrifugation of whole porcine blood at 1500 RPM for 15 min. The inner surfaces of DLSs from each group were punched using a 10 mm biopsy punch (Acuderm Inc., Fort Lauderdale, FL, USA) incubated with 0.5 mL PRP for 1 h, and finally washed twice with PBS. The samples were fixed overnight in 2.5% glutaraldehyde, post-fixed in osmium tetroxide, and dehydrated through a series of graded ethanol solutions. Subsequently, they were coated with a thin layer of gold–palladium and examined using a scanning electron microscope (SEM) (Gemini 560, Oberkochen, Germany).

### 4.5. Re-Endothelialization of Rat Liver Scaffold

Human EA. hy926 endothelial cells (ATCC, Manassas, VA, USA) were cultured in Dulbecco’s modified Eagle’s medium (DMEM) supplemented with 10% FBS and 1% ABAM. The DLS and HEP-DLS were perfused with DMEM media for 30 min for stabilization of the scaffolds prior to re-endothelialization. For re-endothelialization into the vasculature, a total of 2.4 × 107 EA.hy926 cells suspended in 6 mL of the culture media were injected into the scaffold through the PV in two subsequent injections at 15 min intervals. After 3 h of static culture, the scaffolds were perfused with the media within bioreactors using a peristaltic pump at a flow rate of 2 mL/min, in a cell incubator at 37 °C and 5% CO_2_. The culture media were replaced every 48 h, and the recellularized scaffolds were removed on day 7 of in vitro perfusion culture for further analysis. The quantitative analysis of the re-endothelialized vessels was performed by counting 3 different fields per slide from different five lobes.

### 4.6. In Vitro Functional Testing of the Recellularized Scaffold

#### 4.6.1. Cell Viability and Proliferation of the Re-Endothelialized Liver

The Resazurin reduction assay was performed every other day for a duration of 7 days to assess cell viability and proliferation in the re-endothelization liver construct with or without a heparin-immobilized scaffold (n = 3). A 440 μM (10×) stock solution of Resazurin sodium salt (R7017, Sigma Aldrich, St. Louis, MO, US) was prepared in PBS [42]. Working solutions of resazurin (44 μM) were obtained by diluting the stock solution 1:10 in a culture medium. Re-endothelialized livers were perfused with 80 mL of the resazurin working solution at a rate of 2 mL/min for 1 h at 37 °C on days 1, 3, 5, and 7. Photographs of the resazurin-perfused re-endothelialized scaffold were taken at the end of the perfusion. Following incubation, the working solution was replaced with fresh media. The absorbance of the perfused resazurin solution was measured at 570 nm and 600 nm. EC proliferation was presented as the percentage reduction of the resazurin solution and calculated by the formula mentioned in Supplementary S1.

#### 4.6.2. Thrombogenicity of Re-Endothelialized Constructs

To assess the efficiency of endothelialization, both heparin-treated and untreated re-endothelialized liver scaffolds were retrieved from the perfusion chamber after 7 days and then perfused with porcine blood obtained from local slaughterhouse treated with sodium citrate anticoagulant diluted with PBS (1:1) for 1 h. Following blood perfusion, the scaffolds were rinsed with PBS and subjected to immunostaining for platelets using an anti-integrin αIIb antibody (sc-21783, Santa Cruz Biotechnology, Dallas, TX, USA). For the quantification of platelet adhesion, integrin αIIb platelets’ fluorescent intensity was obtained from randomly selected images. Additionally, perfused blood samples from each group were collected for gene expression analysis to evaluate the thrombogenicity using the Hybrid-RTM Blood RNA kit.

### 4.7. Angiogenesis Assay In Vivo

An experimental mice model was used to investigate the effect of HEP-DLS on the angiogenic potential. Following anesthesia and aseptic preoperative procedures, DLSs and HEP-DLSs of 10 mm size scaffolds were implanted into the subcutaneous dorsum of Balb/c mice (n = 4 per group point per time) for 14 and 21 days. At predetermined time points of 14 and 21 days post-implantation, four mice from each group were sacrificed. Comprehensive photographs were taken to visually document the development of new blood vessels surrounding the implanted scaffolds. Samples from each implanted scaffold and the adjacent tissue were collected for histological analyses and IHC staining with an anti-CD31 antibody (ab18298, Abcam, Waltham, MA, USA).

### 4.8. Immunohistochemical Staining

Immunohistochemistry was performed using the ABC detection IHC Kit (Abcam) following the manufacturer’s protocol. Paraffin-embedded sections from re-endothelialized liver scaffolds were deparaffinized, rehydrated, and subjected to antigen retrieval. The slides were incubated with the primary antibodies anti-CD31, 1:500 (ab18298, Abcam) and Ki-67, 1:150 (MA5-14520, Invitrogen, Waltham, MA, USA). For immunofluorescence, the samples were permeabilized with 0.1% Triton X-100 for 15 min and blocked with 2% bovine serum albumin (Sigma-Aldrich) for 45 min. Subsequently, the samples were incubated with the primary antibody anti-integrin αIIb, 1:100 (sc-21783, Santa Cruz Biotechnology). A fluorescent dye-conjugated secondary antibody (goat anti-mouse IgG, Invitrogen) was applied for one hour. Nuclei were stained with DAPI (Sigma-Aldrich). The slides were imaged using an Optinity KCS3-50SS microscope (Korea Labtech, Seongnam, Gyeonggi, Republic of Korea).

### 4.9. TUNEL Assay

To detect apoptotic cells, terminal deoxynucleotidyl transferase-mediated dUTP nick end labeling (TUNEL) was performed on paraffin-embedded sections of the re-endothelialized liver scaffolds using the In Situ Cell Death Detection Kit (Roche Diagnostics, Mannheim, Germany) according to the manufacturer’s protocol. The tissue sections were deparaffinized and digested with proteinase K, then incubated with the enzyme-label solution for one hour at 37 °C. After washing with PBS, the nuclei were stained with DAPI. TUNEL-positive cells were detected using a confocal microscope.

### 4.10. Statistical Analysis

All quantitative data were reported as the mean ± standard deviation. The Student’s *t*-test was used to compare the means. A *p*-value < 0.05 was considered statistically significant.

## 5. Conclusions

In conclusion, this study has demonstrated that the immobilization of heparin to the scaffold efficiently enhances hemocompatibility and promotes EC attachment, survival, and proliferation of rat liver scaffolds. In our study, the heparin-modified scaffold indeed provided a more conducive environment for EC attachment, resulting in improved initial adhesion, accelerated proliferation, and a uniform endothelial lining, which consequently reduced thrombogenicity. Additionally, heparinized scaffolds effectively recruit and localize endogenous angiogenic factors from the host tissue within the scaffold, enhancing new blood vessel formation and demonstrating the angiogenic potential of heparinized scaffolds. This study provides valuable insights into the application of heparin immobilization for re-endothelializing the entire rat liver vasculature, showing significant promise for developing functional bioengineered livers that are suitable for long-term transplantation.

## Figures and Tables

**Table 1:** List of primers and their annealing temperatures used for RT-PCR.

Primer	Sequences	Tm (°C)
Porcine THBS 1	F 5′-TCCTCGTCACATAGGCTGGA-3′ R 5′-ACCACCGGCATAGGTTTTGT-3′	59.6
Porcine TBXAS	F 5′-GCTAGAATCCAAGTCGGCCC-3′ R 5′-CGAGTGAGGGTTGTTGGTGTT-3′	61.5
Porcine PLSCR 1	F 5′-CTAGAAACTGCTGTGGGCCT-3′ R 5′-CATGGGTGCCAGGTTTGAGT-3′	61.5
Porcine GAPDH	F 5′-ACTCACTCTTCTACCTTTGATGCT-3′ R 5′-TGTTGCTGTAGCCAAATTCA-3′	59.6

THBS1, thrombospondin1; TBXAS, thromboxane A synthase; PLSCR1, phospholipid scramblase 1; GAPDH, glyceraldehyde-3-phosphate dehydrogenase transcript variant 1.

## Data Availability

The original contributions presented in the study are included in the article/Supplementary Materials; further inquiries can be directed to the corresponding author.

## Supplementary Materials

The following supporting information can be downloaded at https://www.mdpi.com/article/10.3390/ijms252212132/s1.
