# Supplementary material for: Heparin Immobilization Enhances Hemocompatibility, Re-Endothelization, and Angiogenesis of Decellularized Liver Scaffolds
Source: Int J Mol Sci. 2024 Nov 12;25(22):12132. doi: 10.3390/ijms252212132 (PMC11595110; doi:10.3390/ijms252212132)
Supplement: Supplementary file 1 [file ijms-25-12132-s001.zip › ijms-3290670-SI.pdf]

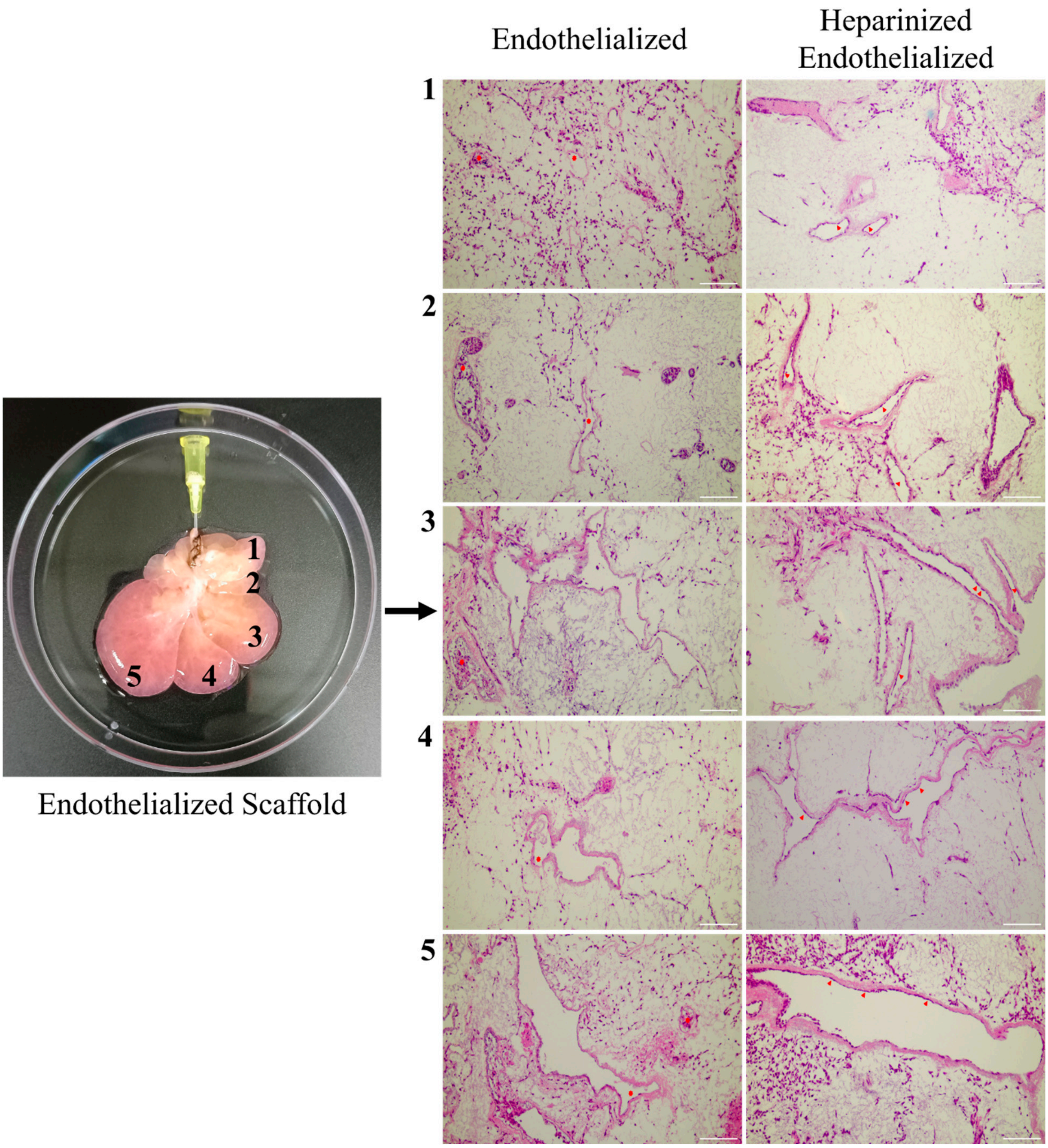

Supplementary Figure S1. Structural characterization of re-endothelialized scaffold. H&E staining of 5 different lobes of re-endothelilialized scaffolds indicated as above (1-5). Heparinized re-endothelialized liver scaffold shows robust attachment of EC to the vasculature, forming a well-defined endothelial cell lining within the blood vessels indicated by red arrows while in endothelialized scaffolds (control) no vessel lining was observed, ECs were clogged in vessels and also dispersed in the parenchymal areas indicated by red stars, (scale bar=100µm).

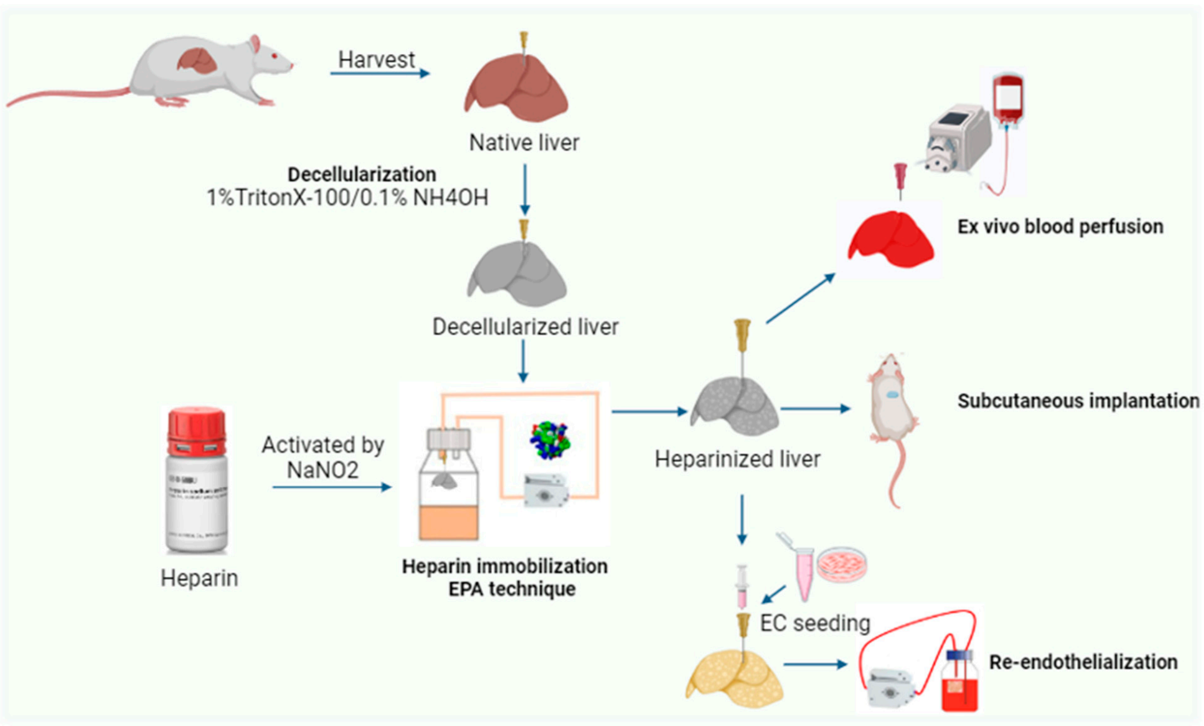

Supplementary Figure S2. The scheme of whole experiment process.

Supplementary S1. Formula for % Reduction of Resazurin sodium salt.

The cell proliferation was presented as the % Reduction of Resazurin sodium salt following the equation below and using the Molar Extinction Coefficient.

% Reduction of Resazurin = (O2 × A1) - (O1 × A2) 100/ (R1 × N2) - (R2 × N1)

O1=Molar Extinction Coefficient of Oxidized Resazurin at 570 nm is 80586

O2=Molar Extinction Coefficient of Oxidized Resazurin at 600 nm is 177216

R1 =Molar Extinction Coefficient of Reduced Resazurin at 570 nm is 155677

R2 =Molar Extinction Coefficient of Reduced Resazurin at 600 nm is14652

A1=Absorbance value of test wells at 570 nm

A2=Absorbance value of test wells at 600 nm

N1=Absorbance value of Negative Control well at 570 nm

N2=Absorbance value of Negative Control well at 600 nm
